# An up-to-date overview of baicalein and its biological and pharmacological activities

**DOI:** 10.17179/excli2025-8559

**Published:** 2025-08-22

**Authors:** Hyeon Ji Yeo, Jung Hun Lee, Sun Sik Kong, Mun Hyoung Ahn, Jiye Rhee, Chang Ha Park

**Affiliations:** 1 Honam National Institute of Biological Resources, 99 Gohadoangil, Mokpo, 587262, Republic of Korea; 2 Department of Digital Healthcare Convergence, Songho University, 210 Namsan-Ro, Hoengseong-Eup, Hoengseong-Gun, Gangwon-Do, Republic of Korea; 3 College of General Education, Namseoul University, 91 Daehak-ro, Seonghwan-eup, Seobuk-gu, Cheonan-si, Chungcheongnam-do 31020, Republic of Korea; 4 Department of Smart Farm, Namseoul University, 91 Daehak-ro, Seonghwan-eup, Seobuk-gu, Cheonan-si, Chungcheongnam-do 31020, Republic of Korea

## ⁯⁯

Baicalein (5,6,7-trihydroxyflavone) is a representative flavone found in *Scutellaria* spp., particularly *Scutellaria* *baicalensis *and *S*.* lateriflora *(Zhu et al., 2024[24]) Baicalein can be biosynthesized from phenylalanine, an initial precursor, with serial enzymatic reactions mediated by phenylalanine ammonialyase, cinnamate 4-hydroxylase, cinnamate-CoA ligase, chalcone synthase, chalcone isomerase, flavone synthase II, and flavone 6-hydroxylase (Yeo et al., 2025[22]). Baicalein is beneficial to human health owing to its antioxidant, anti-inflammatory, anti-cancer, anti-diabetic, antimicrobial, anti-aging, cardioprotective, neuroprotective, respiratory-protective, gastroprotective, liver-protective, and kidney-protective effects (Munjal et al., 2024[15]). In particular, baicalein has received attention as an antitumor agent owing to its high efficacy and low toxicity in the treatment of malignant tumors (Lei et al., 2024[11]). This letter provides an overview of the recent studies conducted to assess the biological and pharmacological properties of baicalein (Table 1(Tab. 1); References in Table 1: Chen et al., 2024[1]; Chi et al., 2025[2]; Dong et al., 2024[3]; Du et al., 2025[4]; Fang et al., 2024[5]; Guo et al., 2024[6]; Hao et al., 2025[7]; Huang et al., 2024[8]; Jin et al., 2024[9]; Lai et al., 2024[10]; Li et al., 2024[13], 2025[12]; Liu et al., 2024[14]; Park et al., 2024[16]; Ren et al., 2024[17]; Wang et al., 2024[18], 2024[19], 2025[20]; Xu et al., 2025[21]; Zhang et al., 2025[23]).

EXCLI Journal 2025;24:1056-1060 – ISSN 1611-2156
Received: May 28, 2025, accepted: June 07, 2025, published: August 22, 2025

| Key message | Reference |
|---|---|
| Baicalein triggers programmed cell death in non-small cell lung cancer by interfering with glutamine uptake via ASCT2 and LAT1, and glutaminase activity, thereby disrupting the mTOR pathway and glutamine metabolism. Both laboratory and animal studies have shown its effectiveness in shrinking tumors and altering metabolic processes, thus suggesting its promising potential as a targeted therapy for glutamine-mTOR-driven cancers. | Li et al., 2025 |
| For colorectal cancer, baicalein promotes ferroptosis by directly suppressing the JAK2/STAT3 pathway, which decreases GPX4 levels and increases lipid peroxidation. Animal studies confirm that baicalein reduces tumor size in a dose-dependent manner through this mechanism. | Lai et al., 2024 |
| In cervical cancer, baicalein increases the effectiveness of cisplatin by inducing cuproptosis, inhibiting the AKT pathway, and boosting cuproptosis-associated proteins (SDHB, FDX1). When combined with cisplatin, baicalein more effectively suppresses tumor growth, thereby providing an additional mechanism to counteract cisplatin resistance. | Jin et al., 2024 |
| In hepatocellular carcinoma, baicalein binds directly to MAPK9, as shown by DNA-programmed affinity labeling, and triggers apoptosis by activating downstream signaling. The use of an MAPK9 inhibitor reverses this effect, thus confirming MAPK9 as a key target for the anticancer action of baicalein. | Wang et al., 2024b |
| In triple-negative breast cancer, baicalein halts disease progression by stabilizing KDM4E, which increases BICD1 expression and promotes PAR1 endocytosis, thus suppressing PAR1 signaling. Loss of KDM4E diminishes the anticancer effect of baicalein, whereas BICD1 overexpression blocks PAR1-driven cancer cell movement and survival. This underscores the importance of the KDM4E-BICD1 axis. | Dong et al., 2024 |
| In oral squamous cell carcinoma, baicalein suppresses metastasis by blocking the ERK/ELK-1/Snail signaling pathway, which reduces cell migration and invasion and reverses EMT markers. When combined with U0126 (ERK inhibitor), the anti-metastatic effects of baicalein are enhanced. | Huang et al., 2024 |
| Baicalein promotes the autophagic breakdown of CD274/PD-L1 by strengthening its interaction with MAP1LC3B/LC3, reducing PD-L1 levels, and enhancing T cell-driven anti-tumor immunity. This mechanism presents a novel strategy for immune checkpoint inhibition by leveraging autophagy. | Hao et al., 2025 |
| In models of acute kidney injury caused by cisplatin or folic acid, baicalein shields renal tubular cells by blocking 12-lipoxygenase (ALOX12), which decreases lipid peroxidation and ferroptosis. Experiments using molecular docking and gene silencing confirm that ALOX12 is a direct molecular target for baicalein, thus supporting its use in treating ferroptosis-induced kidney injuries. | Guo et al., 2024 |
| Baicalein alleviates polycystic ovary syndrome (PCOS) symptoms in a dehydroepiandrosterone-induced rat model by reducing ovarian oxidative stress, lipid peroxidation, and ferroptosis while restoring mitochondrial function and glutathione peroxidase activity. The flavone also improves pregnancy outcomes by modulating ferroptosis-related genes in placental tissue, thereby suggesting its potential as a therapeutic agent targeting ferroptosis pathways in PCOS. | Li et al., 2024 |
| Baicalein alleviates cerebral ischemia-reperfusion injury by upregulating SIRT6 to deacetylate FOXA2, thereby enhancing SLC7A11 expression and suppressing ferroptosis and apoptosis. In MCAO/R mice and OGD/R-induced HT22 cells, baicalein reduced oxidative stress markers (ROS, MDA, $Fe^{2+}$), restored GSH levels, and modulated apoptosis/ferroptosis proteins via the SIRT6-FOXA2 axis. | Fang et al., 2024 |
| In murine hematopoietic cells, baicalein reduces oxidative damage by activating the PDGFRβ-NRF2/AKT pathway, boosting antioxidant defenses, and promoting hematopoietic recovery. The protective effect of baicalein relies on PDGFRβ, as shown in knockout models and molecular interaction studies. | Ren et al., 2024 |

| Key message | Reference |
|---|---|
| Baicalein counters heart failure induced by isoprenaline by restoring mitochondrial dynamics and inhibiting the GRP78/CHOP pathway, which reduces oxidative stress, cell death, and cardiac fibrosis. The efficacy of the flavone is reflected in improved heart function and antioxidant status, thus indicating its potential for heart failure treatment. | Zhang et al., 2025 |
| In lung macrophages, baicalein alleviates oxidative stress by binding to the KEAP1 DGR domain, disrupting the KEAP1-NRF2 interaction, and activating NRF2-dependent antioxidant responses. This action reduces M1 polarization, phagocytosis, and inflammatory cytokine production, thus suggesting a novel therapeutic approach for pneumonia. | Chi et al., 2025 |
| In experimental autoimmune myocarditis, baicalein reduces inflammation by blocking the JAK-STAT1/4 pathway, which limits the polarization of M1 macrophages and Th1 cell differentiation. This reduces TNF-α and IFN-γ secretion and heart muscle cell death. Both *in vivo* and *in vitro* studies show that pathway modulation by baicalein reduces cardiac inflammation and immune-mediated damage. | Wang et al., 2024a |
| Baicalein helps combat acute-on-chronic liver failure (ACLF) by activating the TrKB-CREB1 pathway, which increases TREM2 expression on macrophages and promotes M2 polarization and efferocytosis to dampen inflammation and liver injury. Clinical evidence shows that higher plasma sTREM2 levels are linked to lower inflammation and mortality, thus supporting its value as a prognostic marker. | Chen et al., 2024 |
| Baicalein alleviated chronic itch in a mouse model of allergic contact dermatitis by suppressing the spinal astrocytic STAT3-LCN2 cascade and by reducing scratching behavior and astrocyte activation. *In vivo* and *in vitro* studies confirm baicalein inhibits STAT3 phosphorylation and LCN2 expression, thus offering a potential therapeutic strategy for managing chronic pruritus. | Du et al., 2025 |
| Baicalein attenuates endometriosis progression in mice by reducing proinflammatory cytokine expression and inducing mitochondrial dysfunction. This inactivates MAPK/PI3K signaling, thereby leading to cell death in human endometriotic stromal cells. The anti-inflammatory efficacy of baicalein, demonstrated through *in vivo* and *in vitro* models, positions it as a potential alternative to hormonal therapies with fewer side effects. | Park et al., 2024 |
| In hyperlipidemic acute pancreatitis, baicalein decreases pyroptosis of pancreatic acinar cells by inhibiting the HMGB1/TLR4/NLRP3 pathway and M1 macrophage polarization. Molecular studies show that baicalein binds directly to HMGB1, thus reducing inflammatory cytokines and ROS levels and improving pancreatic injury. | Wang et al., 2025 |
| Baicalein alleviates allergic rhinitis (AR) symptoms in mice by upregulating NR4A1, which transcriptionally represses PRMT1 to inhibit the NF-κB/p65 pathway, thereby reducing inflammatory mediators (IgE, histamine, IL-4/IL-25/IL-33). PRMT1 knockdown reverses NR4A1 deficiency effects in both OVA-induced AR mice and IL-4/IL-13-stimulated nasal epithelial cells, thereby confirming the NR4A1-PRMT1-NF-κB axis as the therapeutic target of baicalein. | Xu et al., 2025 |
| Baicalein alleviates acute lung injury (ALI) by inhibiting the HIF-1α signaling pathway, thereby suppressing glycolysis and reducing pulmonary inflammation and tissue damage. Network pharmacology predictions and experimental validation confirm this mechanism, thus highlighting the role of baicalein in mitigating alveolar damage through metabolic regulation. | Liu et al., 2024 |

ASCT2, alanine/serine/cysteine-preferring transporter 2; LAT11, L-type amino acid transporter 1; mTOR, mammalian target of rapamycin; JAK, Janus kinase; STAT, signal transducer and activator of transcription; GPX, glutathione peroxidase; Akt, protein kinase B; SDHB, succinate dehydrogenase complex iron sulfur subunit B; FDX1, ferreodoxin 1; MAPK, mitogen-activated protein kinase; KDM4E, lysinedemethylase 4E; BICD1, bicaudal D cargo adaptor 1; PAR1, protease-activated receptor 1; ERK, extracellular signal-regulated kinase; ELK-1, E-26-like protein 1; CD274, cluster of differentiation 274; PD-L1, programmed death-ligand 1; MAP1LC3B, microtubule-associated protein 1 light chain 3 beta, LC3, microtubule-associated protein 1A/1B-light chain 3; SIRT, sirtuin; FOXA2, forkhead box protein A2, SLC7A11, solute carrier family 7 member 11; OGD/R, oxygen-glucose deprivation followed by reoxygenation; MCAO/R, middle cerebral artery occlusion/reperfusion; ROS, reactive oxygen species; MDA, malondialdehyde; GSH, glutathione; PDGFRβ, platelet-derived growth factor receptor beta; NRF2, nuclear factor-erythroid 2-related factor 2; GRP78, glucose-regulated protein 78; CHOP, CCAAT/enhancer-binding protein homologous protein; KEAP1, Kelch-like ECH-associated protein 1; DGR, double glycine repeat; Th1, T helper cell type 1; TNF-α, tumor necrosis factor alpha; IFN-γ, interferon gamma; TrKB, tropomyosin receptor kinase B; CREB1, cyclic adenosine monophosphate response element-binding protein 1; TREM2, Triggering receptor expressed on myeloid cells 2; sTREM2, soluble triggering receptor expressed on myeloid cells 2; LCN2, lipocalin 2; PI3K, phosphoinositide 3-kinase; EGFR, epidermal growth factor receptor; HMGB1, high mobility group box 1;

TLR4, toll-like receptor 4; NLRP3, nucleotide-binding domain, leucine-rich–containing family, pyrin domain–containing-3; NR4A1, nuclear receptor subfamily 4 group A member 1; PRMT1, protein arginine methyltransferase 1; NF-κB, nuclear factor kappa B; IgE, immunoglobulin E; IL, interleukin; OVA, ovalbumin; HIF-1α, hypoxia-inducible factor 1 alpha; CCDC88A, coiled-coil domain containing 88A; AMPK, adenosine monophosphate-activated protein kinase; GLP-1, glucagon-like peptide 1; CaMKII, $Ca^{2+}$/calmodulin-dependent protein kinase II; GLUT4, glucose transporter type 4; MyD88, myeloid differentiation primary response; VEGF, vascular endothelial growth factor; PlGF, placental growth factor; MMP9, matrix metalloproteinase 9; CXCL2, C-X-C motif chemokine ligand 2; DPP-4, dipeptidyl peptidase 4; PGC-1α, peroxisome proliferator-activated receptor gamma coactivator 1-alpha.

## *Conflicts of interest*

The authors declare no conflict of interest.

## Declaration

### Acknowledgments

Funding for this paper was provided by Chungcheongnam-do Regional Innovation System for Education (RISE) Project.

### Conflicts of interest

The authors declare no conflict of interest.

## Figures and Tables

**Table 1:**
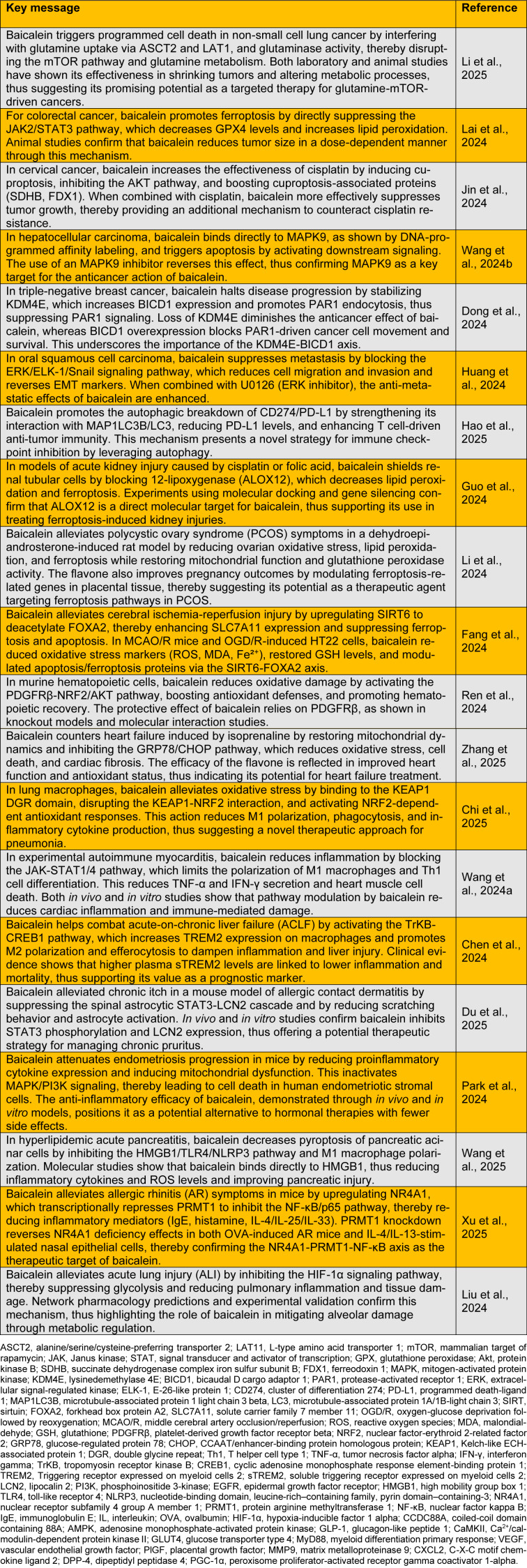
The biological and pharmacological activities of baicalein according to recent studies
